# Anger and Sadness Expressions Situated in Both Positive and Negative Contexts: An Investigation in South Korea and the United States

**DOI:** 10.3389/fpsyg.2020.579509

**Published:** 2021-01-13

**Authors:** Sunny Youngok Song, Alexandria M. Curtis, Oriana R. Aragón

**Affiliations:** ^1^Department of Marketing, Wilbur O. and Ann Powers College of Business, Clemson University, Clemson, SC, United States; ^2^School of Marketing and International Business, Spears School of Business, Oklahoma State University, Stillwater, OK, United States

**Keywords:** facial expressions, motivation and affect, Dimorphous, expression, emotion

## Abstract

A formidable challenge to the research of non-verbal behavior can be in the assumptions that we sometimes make, and the subsequent questions that arise from those assumptions. In this article, we proceed with an investigation that would have been precluded by the assumption of a 1:1 correspondence between facial expressions and discrete emotional experiences. We investigated two expressions that in the normative sense are considered negative expressions. One expression, “anger” could be described as clenched fists, furrowed brows, tense jaws and lips, the showing of teeth, and flared nostrils, and the other “sadness” could be described as downward turned mouths, tears, drooping eyes, and wrinkled foreheads. Here, we investigated the prevalence, understanding, and use of these expressions in both positive and negative contexts in South Korea and the United States. We found evidence in both cultures, that anger and sadness displays are used to express positive emotions, a notion relevant to Dimorphous Theory. Moreover, we found that anger and sadness expressions communicated appetitive feelings of wanting to “go!” and consummatory feelings of wanting to “pause,” respectively. There were moderations of our effects consistent with past work in Affect Valuation Theory and Display Rule Theory. We discuss our findings, their theoretical relevance, and how the assumptions that are made can narrow the questions that we ask in the field on non-verbal behavior.

## Introduction

A formidable challenge for the research in non-verbal behavior may lie in the assumptions that have been made concerning a correspondence between facial expressions and basic emotions e.g., the “anger face” corresponds to feelings of anger, smiles correspond to happy feelings, or tearful crying corresponds to feelings of sadness (Ekman and Friesen, 1971, 1986; Izard, 1971; Ekman, 1972). Such assumptions about correspondence between facial movements and specific emotional experiences were empirically based, and also intuitive because there *does* seem to be a tacit agreement of the normative, almost definitional understanding of expression-experience correspondence observable in our world today (Russell, 1994). For example, elementary school rooms feature charts to teach children expressions and their corresponding discrete emotions, and there was a relatively instantaneous worldwide adoption of emoji faces (Danesi, 2017), possibly because we already had an implicit consensus of what the basic emoji expressions meant.

However, such an assumption of a 1:1 correspondence between facial expressions and experience is upended when considering how these expressions are actually used in real life. Our literature (for example, Fernández-Dols and Ruiz-Belda, 1995; Carroll and Russell, 1996; Fernández-Dols et al., 1997; Aviezer et al., 2008, 2012; Fernandez-Dols and Crivelli, 2013; Aragón et al., 2015; García-Higuera et al., 2015; Durán et al., 2017; Aragón and Bargh, 2018; Aragón and Clark, 2018) and lives abound with examples of violations of this supposed correspondence, e.g., the happy tears upon the birth of one’s child, the seemingly violent rage across a soccer field upon winning, a smile when embarrassed, and the tooth baring growl at a cute little baby. There might be a definitional understanding of a correspondence between expression and discreet emotions, but that appears to be separate and apart from how expression and discrete emotions correspond in real life.

In this article we proceed to report on an investigation that would have been precluded by the assumption of a 1:1 correspondence between expression and experience. This investigation put aside the definitional understanding of two expressions that, in the normative sense are considered negative expressions, but within experiments with Western samples have been found to violate normative correspondence when interpreted in context. One expression could be described as clenched fists, furrowed brows, tense jaws and lips, the showing of teeth, and flared nostrils, and the other described as downward turned mouths, tears, drooping eyes, and wrinkled foreheads. For a lack of better terminology, we will refer to these physical displays as “anger” and “sadness” expressions, respectively. Our primary aim was to understand how anger and sadness expressions are interpreted in both positive and negative contexts, and if people report to use anger and sadness expressions in both positive and negative contexts, in South Korea and the United States.

### Anger and Sadness Expressions as Dimorphous Expressions of Emotion

Previous research has shown that when anger and sadness expressions are situated within positive contexts the majority of observers show consensus in their interpretation of the expressions as representing predominantly positive—not negative experiences (Fernández-Dols and Ruiz-Belda, 1995; Aviezer et al., 2012; Aragón et al., 2015; Aragón, 2016, 2017; Wenzler et al., 2016; Aragón and Bargh, 2018; Aragón and Clark, 2018). This finding that anger and sadness expressions can be associated with predominantly positive emotions has been consistent whether the expressions arose within participants themselves during emotionally evocative situations (Aragón et al., 2015; Aragón, 2017), the expressions were presented to participants to probe for reflection of their own past experiences (Aragón and Bargh, 2018), or when participants were asked to interpret what those expressions might represent (Aviezer et al., 2008; Aragón, 2016, 2017, 2020; Aragón and Bargh, 2018; Wenzler et al., 2016). These patterns were consistent whether anger and sadness expressions were pulled from photographs of real-life contexts (Aragón and Bargh, 2018), created through trained actors (Aragón, 2017; Aragón and Clark, 2018), were photographs of anger and sadness classified through facial action coding (Karolinska directed emotional faces; Lundqvist et al., 1998 as used in Aragón and Bargh, 2018), and whether experiences had been presented through static photographs, narrative accounts, or dynamic video displays.

These types of expressions, in which the normative interpretation and the contextual interpretation are opposing in valence, were termed “dimorphous” (Aragón et al., 2015). Expressions that are called dimorphous share systematic features. For one, dimorphous expressions are context dependent for accurate interpretation, i.e., an overjoyed woman who displays a downward turned mouth, flowing tears and wrinkled brow would be interpreted as experiencing a negative emotion if viewed out of context (most likely because of our implicit understanding), but is instead read as experiencing positive emotion in the context receiving her Olympic gold medal (Fernández-Dols and Ruiz-Belda, 1995; Aviezer et al., 2012; Aragón and Clark, 2018). This highlights the idea that emotional experiences and facial movements do not have a 1:1 correspondence. Dimorphous expressions are uncontrolled and spontaneous, i.e., not forced, not produced sarcastically to make a point, not in service of emotional labor, or masks to hide one’s true feelings. Dimorphous expressions are displays that unfold over the course of an emotional event, e.g., “He was smiling, and he was so happy he even cried.” The two expressions alternate or may combine at times during the emotional event. The term dimorphous was chosen to reflect this unfolding real-life dynamic of two expressions arising from a singularly valenced emotional experience.

Dimorphous expressions are by definition the experience of a singularly valenced emotional experience, which makes them distinct from the hypothesized simultaneous experience of positive and negative emotions as described in mixed emotions (Larsen et al., 2001), and from sequentially experienced positive and negative emotions (Carrera and Oceja, 2007; Russell, 2017). In an experiment in which participants watched a predominantly positive heart-warming story in which the hero lived a long and happy life, Aragón (2017) demonstrated that dimorphous expressions represented a singularly valenced appraisal (good things took place, and bad things did not take place) that produced a singularly valenced experience of emotion (I feel good feelings, and I do not feel bad feelings), resulting in the display of two physical expressions (I smiled and I cried) over the course of participants’ own emotional experience. Those physical displays were attributed by participants to their own singularly valenced feelings (I smiled and cried because of the positive emotions I was feeling). In contrast, when participants were assigned randomly to view the same heartwarming story but in this case told that the hero had died, both positive and negative emotions were evident, as participants made two appraisals opposing in valence (both good and bad things took place), associated with two emotional experiences opposing in valence (I feel good and bad feelings), which then resulted in the display of two physical expressions over the course of an emotional episode (I smiled and I cried), that were attributed to both positive and negative feelings (I smiled and cried because of the positive and negative emotions I was feeling). These findings were consistent with self-report measures and with implicit measures of positive and negative affect.

Additionally, dimorphous expressions can be of a singular “flavor” of emotion, e.g., crying when feeling intensely relieved, but dimorphous expressions are also consistent with the idea that expressors can experience a blend of positive emotions, for example feeling both relieved and joyous when crying (Smith and Ellsworth, 1987). In dimorphous expression research, emphasis has been placed less so on which flavor of emotion is displayed, and more so upon the overall valence of the experience. For example, the expression of a positive emotion through an anger display may represent pride (Aragón and Bargh, 2018), victory (Aviezer et al., 2008), excitement (Aragón, 2016), or even overwhelming feelings of care when regarding something adorably cute (Aragón et al., 2015). Dimorphous research has focused on understanding the correspondence between the normatively understood valence of expression and the valence of the actual experience. The precise flavor of emotional experience has been of less importance for this research focus (for discussion see, Aragón and Bargh, 2018).

Researchers who have measured both emotional experiences and what we refer to as dimorphous expressions, consistently note that those who express emotion dimorphously describe their emotions as intense (e.g., Fredrickson and Levenson, 1998; Bonanno and Keltner, 2004; Ansfield, 2007; Aragón et al., 2015; Aragón, 2016, 2017; Aragón and Bargh, 2018; Aragón and Clark, 2018). Additionally, researchers note that dimorphous expressions are interpreted by raters, onlookers, or judges as highly intense (e.g., Fernández-Dols and Ruiz-Belda, 1995; Aviezer et al., 2012; Aragón and Clark, 2018), and that the situations in which these expressions arise are themselves judged to be intense in nature (e.g., Fernández-Dols et al., 2010; Wenzler et al., 2016). It is possible that the alternating display between the normatively corresponding and non-corresponding expression is a function of a fluctuating intensity of emotion. For example, as one wins an award, she may predominantly smile, however, momentarily cry when she is hit with a new wave of highly intense feelings.

When trying to understand the functional nature of these expressions, it is important to understand what they discreetly communicate. As discussed above, anger and sadness expressions have been found to be poor indicators of emotional valence because they communicate positive emotions when situated in positive contexts, and negative emotions when situated in negative contexts. Both expressions signal intense experiences, and thus do not discriminate well from each other in the aspect of intensity of experience. And, anger and sadness expressions, particularly within positive contexts can relay any of a variety of flavors of emotion, e.g., anger and sadness expressions can both signal feelings of pride, or adoration, or victory. Since anger and sadness expressions do not discriminate in the aspects outlined above, here we describe what anger and sadness expressions have been found to distinctly communicate across both the positive and negative situations.

### Activation-Type Dimensions Associated With Anger and Sadness Expressions

Researchers have previously introduced useful theories about activation-type dimensions of emotion, i.e., activation and deactivation (Russell, 2003), excitement and calm (Mogilner et al., 2012), excited and peaceful happiness (Tsai et al., 2006), high and low states of action readiness to engagement (Frijda et al., 1989), high and low states of arousal (Russell, 1980; Feldman Barrett, 1998), dominance and submissiveness (Bradley and Lang, 1994), promotion and prevention focus (Higgins, 1997), and appetitive and consummatory aspects of pleasure (Berridge and Robinson, 2003). In a quest to understand what angry and sadness expressions might consistently communicate, Aragón and Bargh (2018) tested these overlapping constructs in a series of experiments to see which, if any, would be associated distinctly with anger or sadness expressions, whether situated in positive or negative contexts.

Over a series of studies, the constructs of high arousal (feelings associated with words such as excited, active, and alert) and low arousal (words such as calm, depleted, and sleepy) were differentiated for anger and sadness i.e., anger expressions were viewed as more so high arousal, and sadness expressions were viewed as more so lower arousal, but this was only true when those expressions were situated in negative contexts. When asked if a winning athlete who showed an anger expression was excited, participants indicated yes, she was. But also, when asked if a winning athlete who cried was excited, again participants would indicate yes. And when given an open text box to describe how they interpreted the sadness expression in a positive context, participants would describe, “She’s excited. She just needs to stop for a minute,” or “She was just overwhelmed and needed to pause a minute.” A similar pattern emerged for the aspects of dominance and submissiveness, which again only discriminated between anger and sadness expressions in negative contexts. These findings were true whether the paradigm tested for participants’ own experiences with anger and sadness expressions, or participants’ interpretations of what others were feeling when those others displayed anger or sadness expressions.

Over many iterations only one conceptualization within the overlapping activation-type constructs showed a consistent discrimination between the anger and sadness expressions across both negative and positive contexts, those were the fundamental motivational orientations of feelings of “wanting to go” and “wanting to stop.” Anger expressions that arose in positive or negative contexts represented and communicated positive and negative emotional experiences, respectively, that were imbued with antsy feelings of wanting to go, move or accelerate as put forth by Berridge and Robinson (2003) in the concept of appetitive pursuit. In contrast, sad expressions that arose in both positive and negative contexts, represented and communicated positive and negative emotional experiences, respectively, that were imbued with spent feelings of wanting to pause, stop, or be still, akin to the concept of consummatory states in which one pauses from pursuit (Berridge and Robinson, 2003).

Even more important to the possible reason why dimorphous expression might exist at all, i.e., “Why don’t smiles seem to suffice in these intensely positive moments?” dimorphous expressions appear to provide information about the expressers’ appetitive and consummatory orientations with a specificity that smiles did not. This is supported by the fact that positive emotions expressed through anger and sadness displays have also been found to impact inferences about expressers’ experiences and future product preferences. For example, people who expressed they were so happy as to “yell ‘YES!”’ were deemed to be more likely to prefer an action vacation package and people who expressed that they were so happy they cried were deemed more likely to prefer a relaxation vacation package. These orientations even imbued participants’ inferences about the types of products that were in use when the expressions had arisen, i.e., drivers with anger expressions were driving zippy sports cars, and drivers who displayed joyous tears were driving luxury sedans (Aragón, 2016). In American samples to date, anger and sadness expressions have provided robust signals to onlookers about the expressers’ feelings of wanting to go or wanting to pause, respectively. Considering the choreography of social interactions, such information would seem vital, particularly in situations in which emotions are intense.

### Overview

This study included respondents from both the United States and South Korea. In the experimental portion of the study, participants were assigned randomly to view people donning anger, smiling, or sadness expressions situated within short vignettes. There were five vignette themes designed to elicit different flavors of emotion. Each of the five vignette themes was tailored to feature a positive and negative version within the same theme, e.g., someone fulfills a lifelong dream, versus someone fails to fulfill a lifelong dream. Every participant saw a total of 10 vignettes (5 themes × 2 versions) that displayed models who varied in gender, background (Asian, Black, Latino/a, and White), age—some being apparently college-aged, and some appearing to be in their early to mid-thirties, and who showed no apparent signals of authority, e.g., in plain clothing against plain backgrounds. Our dependent variables of interest were participants’ interpretations of the expressers’ emotional intensity, experience (positive and negative), and the expressers’ appetitive and consummatory motivational orientations. Additionally, to understand prevalence of these expressions cross-culturally, we also asked participants if they had seen, known someone, or had themselves used the depicted expression in a similar context. Separated in time from the experimental portion but in the same research session we also collected individual difference measures of participants’ tendencies to express emotion dimorphously (Dimorphous Expression Questionnaire, Aragón et al., 2015).

#### Central Study Aims

Our central question pertained to the existence of dimorphous expressions across these two cultures and that was to be addressed through the experimental design, and the individual difference measure. We reasoned that if dimorphous expressions existed in South Korea as they have been observed in the United States, that in the experimental portion South Korean participants would interpret anger and sadness expressions in positive contexts much as Americans do, as intense positive, but not negative emotional experiences. And when asked if they had seen, known, or themselves used such an expression of anger or sadness in a positive context, agreement that they had would be further evidence for dimorphous expressions. Additionally, we expected that the individual difference measures of dimorphous expressions could capture the existence of dimorphous expressions.

H1: We predicted that when anger, smiling, and sadness expressions were situated in positive contexts they would be interpreted as representing predominantly positive emotional experiences, and when situated in negative contexts they would be interpreted as representing predominantly negative emotional experiences.
H2: We predicted that anger and sadness expressions would communicate appetitive and consummatory orientations, respectively, and smiling expressions would not clearly communicate either appetitive or consummatory orientations.
H3: We predicted that anger and sadness expressions would communicate more intense emotional experiences than smiling expressions.
H4: We predicted that participants in both the South Korea and the United States would self-report the use of dimorphous expression.

#### Three Cross-Cultural Considerations for Our Hypotheses

One consideration was that people from Western, individualistic contexts strive to maximize positive and minimize negative emotions, whereas those from Eastern, collectivist contexts instead value experiencing both positive and negative emotions. Thus whether conceptualized as a mixed experience of emotions (Larsen et al., 2001) or sequentially experienced emotions (Carrera and Oceja, 2007), people from Eastern contexts have more experiences with a combination of both positive and negative emotions than people from Western contexts (Sims et al., 2015). These cultural differences in the presence of both positive and negative emotions apply more so in positive than negative settings (Kim et al., 2014), presumably because people from Eastern contexts see a mix of both positive and negative emotions as creating a tempered balance or harmony during positive events, and those from Western contexts strive to feel more purely positive. Therefore, we considered the following possibility:

H5: We left open the possibility that participants from South Korea might not express emotions dimorphously, and as such might not interpret anger and sadness expressions situated in positive contexts as representing predominantly positive emotions.

A second consideration involved previous work in affect valuation theory that has found that North American (United States and Canada) students idealize high arousal positive affect more so than low arousal, and East Asian (Chinese, Japanese, and Korean) students more so idealize low arousal positive affect over high arousal (Tsai et al., 2006). Idealized affect in turn predicts typically experienced affect ostensibly because the experiences one might choose to engage in could differ by how one would like to feel during the experience. These experience selections are reflected in the products (Chim et al., 2018; Park et al., 2020), professional services such as doctors (Sims et al., 2018), and leaders (Tsai et al., 2016) that are preferred cross-culturally. Higher arousal versions are preferred in Western cultures and lower arousal versions preferred in Eastern cultures. Therefore, one might expect similar patterns in the prevalence of the expression of positive emotion as anger and sadness expressions, respectively, because anger expressions are related to appetitive states that conceptually overlap with high arousal, and sadness expressions are related to consummatory states that conceptually overlap with low arousal.

H6: We predicted that South Korean participants would report using more sadness than anger expressions and United States participants would report using more anger than sadness expressions to communicate positive emotions.

A third consideration was in regard to how display rules might differ between these cultures. Individualistic cultures are more so focused on the development of the self (Markus and Kitayama, 1991), personal goals (Yamaguchi, 1994), and the expression of emotion (Butler et al., 2007; Matsumoto et al., 2008b). In contrast, collectivist cultures are more so focused on the development of the in-group, the in-group’s goals, place a lesser value on the expression of an individual’s emotions (Matsumoto et al., 2008a), particularly anger and sadness expressions (Matsumoto, 1990; Safdar et al., 2009), and encourage the suppression, i.e., holding back (Gross, 2001) of emotion, or the masking of negative emotions with smiles (Friesen, 1972; Ekman and Friesen, 1982; Matsumoto and Kupperbusch, 2001; Rychlowska et al., 2017).

H7: We predict that our South Korean sample would report fewer expressions of emotion than our American sample, with the exception of smiling to mask negative experiences.

## Materials and Methods

### Participants

Students from an American State University and South Korean Universities were recruited through classroom announcements and through university electronic bulletin boards to participate in this approximate 20-min study for either course credit or approximately $5 US dollars during the fall of 2019. Participants who participated for cash compensation were paid via an online application. Students participating for course credit were compensated through a department subject pool.

Sample size was estimated with the experimental design’s between factors in mind: 2 (country) × 2 (gender) × 3 (expression) = 12 cells at 50 participants per cell estimated in Aragón and Bargh (2018). This calculation rendered a goal to recruit 600 participants. Our sample for the dimorphous expression questionnaire (*N* = 659) included data from all non-international (i.e., native to each respective country) participants who passed attention checks and completed through the individual difference measure of dimorphous expressions located near the beginning of the survey: South Korean (*N* = 305, 132 men, *M*_age_ = 20.16, *SD* = 2.55, SK age- corrected for cultural differences in numerical assignment) and American (*N* = 354, 169 men, *M*_age_ = 20.03, *SD* = 2.05). There was attrition (*n* = 75) during this 20-min survey. Our sample for the experimental portion which occurred about 10 min into the survey included all who completed through that portion (*N* = 584).

### Materials and Procedure

Data were collected as part of a larger investigation. See Supplementary Appendix A for study details. All materials were developed in English, translated into South Korean (by Song), and back translated into English by paid interpreters. Originals and backtranslations were then compared for meaning (by Aragón). When discrepancies arose, adjustments to the translated version were made (Brislin, 1970). This process took three iterations. We ran a pilot of this study in the summer of 2019 with 66 participants from South Korea. Only a few minor wording changes were made after the pilot study. The findings from the pilot and this study are nearly identical.

#### Experimental Paradigm

Participants were assigned randomly to consider either anger, smiling, or sadness expressions, displayed within five vignette themes, that were designed with a both a positive and negative version (10 trials in total). The vignette themes were selected to reflect the instances in which participants have previously reported to express dimorphously (Aragón et al., 2015; Aragón, 2016; Aragón and Bargh, 2018; Aragón and Clark, 2018), such as when (1) a person has the opportunity to fulfill a lifelong dream, (2) a person views a beautiful nature scene, (3) a person accomplishes a big life goal after a long struggle to succeed, (4) a person is reunited with family after a long absence, and (5) a fan is able to see a favorite celebrity. Negative versions also were created for each of these themes, please see Supplementary Appendix B for all scenarios.

We chose five themes so that our effects would not be bound to a single “flavor” of emotion, because we would be able to demonstrate for example, that sadness expressions can communicate positive emotions that would come about in a beautiful nature scene as well as would come about in accomplishing a big life goal. To have confidence that the positive events were considered predominantly positive, and the negative events were considered predominantly negative, an independent sample of participants (*N* = 61 online; 38% women, *M*_age_ = 24.41) validated that the “positive” vignettes were considered predominantly positive, and not negative, and the “negative” vignettes were considered predominantly negative, and not positive. There were no interactions of vignette, meaning all of the “positive” vignettes were interpreted as equally positive, and equally not negative, and all of the “negative” vignettes were interpreted as equally negative, and equally not positive. See Supplementary Appendix B for means.

**FIGURE 1:**
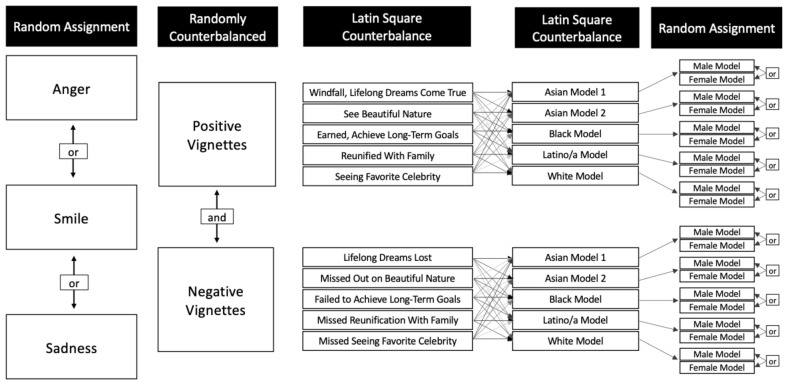
In the experimental portion, participants were assigned randomly to view models with either an anger, smiling, or sadness expression. Counterbalancing ensured that all participants responded to five positive and five negative vignettes, with Asian, Black, Latino/a, and White models, that were either male or female.

Inserted just below the wording of each vignette was a photograph of a man or woman (randomly selected) of apparent Asian, White, Black, or Hispanic/Latinx background (counterbalanced). All vignette, expression, gender, and model pairing combinations were presented an equal number of times. This design made it less likely that our observed effects would be due idiosyncratic factors of a model’s gender, the vignette type, a model’s ethnicity, or a model’s particular anger, smiling, or sadness expression. See Figure 1.

**FIGURE 2:**
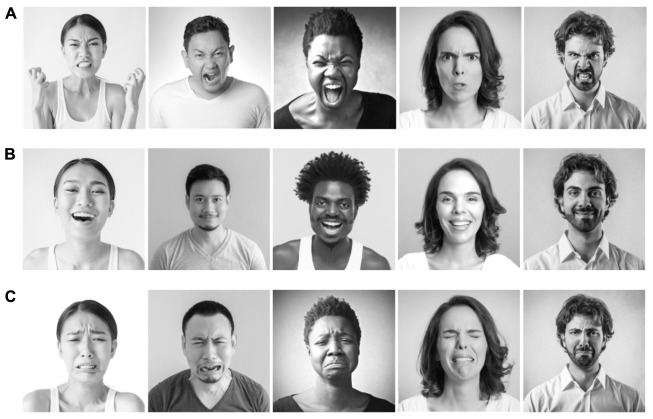
Pictured are examples from the 30 photographs [10 models each depicting anger **(A)**, smiling **(B)**, and sadness **(C)**] used for our experiment. Standard content license was purchased with signed model release on file at Shutterstock.

The photographed models were purchased (Shutterstock, 2019) and independently validated (*N* = 105, online sample; 37% women, *M*_age_ = 36.70) to give us confidence that what we considered normative anger, smiling, and sadness expressions were actually understood normatively as representing anger, happiness, and sadness, respectively. In a forced-choice paradigm, participants viewed each of our modeled expressions and selected from labels of happy, angry, sad, surprised, fearful, or disgusted. This methodology has been used by basic emotion researchers, and we considered it appropriate to capture a normative understanding of the expression - experience correspondence (as described in Russell, 1994). In our stimuli validation study 73.1% of respondents considered our “angry” models to be angry, 93.7% considered our “smiling” models to be happy, and 76.5% considered our “sadness” models to be sad. Asian models were overrepresented in our design (4 Asian, 2 Black, 2 Latino/a, and 2 White models) because we took into account that our South Korean participants would come from a less diverse context (Fearon, 2003). Without this adjustment, South Korean participants would have evaluated a greater percentage of models who appeared to be outgroup members than would have American participants. See Figure 2 for examples of the expressions used.

Following each of the 10 vignettes, participants indicated their inferences about the expressers’ emotions with, “He (or she) is feeling _______” positive valence: “strong positive emotions” and “good emotions,” and for negative valence: “strong negative emotions” and “bad emotions.” We also asked about the motivational orientations of the expresser with, “He (or she) is feeling like he (she) wants to _______” appetitive items: “go, go, go!” and “get moving,” and consummatory items “stop for a moment” and “be still for a moment.” We also asked the intensity of the emotion perceived “He (she) is feeling intense emotions.” Then, to assess the prevalence of anger, smiling and sadness expressions for these vignettes for each culture, three items asked participants if they had seen, known someone, or had themselves used “this type of expression in a positive (or negative) event.” Response options were 1 = *Strongly Disagree*, 2 = *Disagree*, 3 = *Somewhat Disagree*, 4 = *Somewhat Agree*, 5 = *Agree*, and 6 = *Strongly Agree*. See Table 1, for descriptive statistics.

**TABLE 1:** Descriptive statistics for the seen, known, and used the expression items.

			Positive vignettes				Negative vignettes			
	Vignette	Expression condition	South Korea		United States		South Korea		United States	
			% Agreed	Mean (SD)	% Agreed	Mean (SD)	% Agreed	Mean (*SD*)	% Agreed	Mean *(SD)*
Seen % who agreed or strongly agreed (5 or 6) and mean scores (*SD*)	Fortune	Anger-like	36.7	3.54 (1.69)	51.9	4.21 (1.49)	16.0	2.71 (1.57)	63.9	4.52 (1.25)
		Smile	55.4	4.43 (1.27)	72.9	4.92 (0.80)	36.3	3.86 (1.33)	29.5	3.64 (1.36)
		Sadness-like	30.2	3.52 (1.57)	57.5	4.38 (1.37)	11.9	2.69 (1.49)	72.8	4.84 (1.01)
	Awe	Anger-like	22.9	3.05 (1.60)	31.8	3.46 (1.48)	16.5	2.97 (1.48)	57.4	4.42 (1.18)
		Smile	54.7	4.49 (1.10)	70.5	4.80 (0.97)	39.0	4.04 (1.29)	23.6	3.46 (1.31)
		Sadness-like	20.5	3.25 (1.41)	40.4	3.87 (1.48)	16.9	2.76 (1.57)	65.2	4.64 (1.14)
	Pride	Anger-like	36.7	3.74 (1.67)	61.7	4.57 (1.28)	13.4	2.61 (1.55)	68.2	4.84 (1.06)
		Smile	60.8	4.65 (0.91)	70.3	4.88 (0.95)	33.8	3.77 (1.38)	31.0	3.60 (1.39)
		Sadness-like	32.6	3.45 (1.65)	53.5	4.35 (1.39)	17.9	2.79 (1.62)	73.0	4.81 (1.08)
	Interpersonal	Anger-like	28.4	3.36 (1.60)	45.8	4.07 (1.38)	16.8	2.68 (1.53)	67.6	4.70 (1.10)
		Smile	58.4	4.57 (1.04)	67.4	4.70 (0.95)	34.2	3.65 (1.47)	29.7	3.58 (1.36)
		Sadness-like	34.9	3.86 (1.51)	67.5	4.78 (1.20)	11.8	2.49 (1.60)	68.7	4.68 (1.20)
	Ecstatic	Anger-like	37.4	3.62 (1.71)	42.6	3.94 (1.59)	12.4	2.66 (1.48)	66.7	4.56 (1.15)
		Smile	55.4	4.47 (1.11)	70.5	4.83 (0.89)	38.0	3.80 (1.35)	34.6	3.80 (1.32)
		Sadness-like	32.2	3.69 (1.46)	56.1	4.43 (1.30)	20.2	2.70 (1.61)	65.2	4.63 (1.20)
	Total, % agreed in at least 1 vignettes, mean (*SD*)	Anger-like	63.1	3.43 (1.24)	83.3	4.05 (0.94)	41.6	2.76 (1.10)	89.8	4.60 (0.83)
		Smile	84.6	4.51 (0.75)	89.9	4.82 (0.69)	70.4	3.84 (1.00)	58.5	3.63 (1.01)
		Sadness-like	65.2	3.54 (1.05)	84.2	4.36 (1.00)	46.0	2.70 (1.12)	89.6	4.72 (0.86)
Known % who agreed or strongly agreed (5 or 6) and mean scores (*SD*)	Fortune	Anger-like	38.8	3.52 (1.69)	45.4	4.04 (1.50)	16.2	2.74 (1.54)	54.6	4.40 (1.24)
		Smile	52.0	4.37 (1.32)	68.2	4.75 (0.94)	35.0	3.88 (1.34)	25.6	3.55 (1.32)
		Sadness-like	29.1	3.50 (1.63)	53.1	4.30 (1.38)	13.1	2.71 (1.57)	68.4	4.72 (1.04)
	Awe	Anger-like	19.8	2.97 (1.54)	27.8	3.43 (1.44)	17.3	2.89 (1.48)	56.5	4.33 (1.20)
		Smile	52.0	4.39 (1.06)	72.9	4.81 (0.91)	33.3	4.00 (1.28)	24.4	3.47 (1.31)
		Sadness-like	18.0	3.19 (1.40)	39.5	3.88 (1.43)	9.6	2.52 (1.43)	60.0	4.57 (1.08)
	Pride	Anger-like	41.8	3.84 (1.65)	58.3	4.43 (1.29)	16.5	2.66 (1.57)	65.1	4.70 (1.10)
		Smile	56.8	4.57 (1.09)	71.9	4.87 (0.89)	33.8	3.82 (1.33)	24.8	3.57 (1.30)
		Sadness-like	27.9	3.49 (1.64)	55.3	4.42 (1.30)	17.9	2.79 (1.63)	71.3	4.71 (1.08)
	Interpersonal	Anger-like	29.8	3.23 (1.66)	42.6	3.89 (1.43)	18.9	2.67 (1.54)	61.1	4.62 (1.11)
		Smile	46.8	4.32 (1.15)	66.4	4.66 (1.03)	31.6	3.53 (1.43)	25.0	3.49 (1.36)
		Sadness-like	37.2	3.90 (1.49)	67.5	4.73 (1.22)	11.8	2.52 (1.58)	68.7	4.65 (1.16)
	Ecstatic	Anger-like	31.3	3.37 (1.24)	42.6	3.94 (0.93)	16.7	2.63 (1.56)	60.2	4.49 (1.16)
		Smile	52.7	4.40 (0.79)	62.8	4.76 (0.64)	32.5	3.64 (1.35)	25.2	3.69 (1.26)
		Sadness-like	34.5	3.54 (1.06)	59.6	4.37 (0.97)	18.8	2.62 (1.64)	62.6	4.53 (1.19)
	Total, % agreed in at least 1 vignettes, mean (*SD*)	Anger-like	64.1	3.42 (1.70)	79.6	3.92 (1.60)	46.5	2.77 (1.11)	87.0	4.50 (0.87)
		Smile	82.1	4.43 (1.17)	93.8	4.71 (0.95)	70.4	3.78 (0.88)	59.2	3.57 (0.93)
		Sadness-like	65.3	3.70 (1.53)	82.5	4.50 (1.14)	43.7	2.63 (1.09)	88.7	4.64 (0.82)
Used % who agreed or strongly agreed (5 or 6) and mean scores (*SD*)	Fortune	Anger-like	17.3	2.85 (1.52)	38.0	3.72 (1.59)	12.0	2.38 (1.48)	49.1	4.11 (1.36)
		Smile	45.3	4.04 (1.50)	64.3	4.67 (0.94)	32.5	3.59 (1.46)	20.2	3.18 (1.38)
		Sadness-like	14.0	2.86 (1.47)	27.2	3.40 (1.41)	8.3	2.32 (1.38)	57.0	4.37 (1.22)
	Awe	Anger-like	10.4	2.45 (1.38)	22.2	3.00 (1.49)	5.1	2.27 (1.30)	45.4	4.05 (1.27)
		Smile	41.3	4.05 (1.37)	59.7	4.55 (1.03)	26.9	3.64 (1.31)	15.0	3.07 (1.29)
		Sadness-like	10.1	2.61 (1.38)	21.9	3.07 (1.49)	7.2	2.31 (1.38)	41.7	3.98 (1.41)
	Pride	Anger-like	28.6	3.27 (1.67)	43.5	4.06 (1.47)	7.2	2.16 (1.38)	47.7	4.27 (1.32)
		Smile	45.9	4.26 (1.19)	60.2	4.65 (1.02)	27.3	3.48 (1.39)	18.6	3.19 (1.35)
		Sadness-like	17.4	2.97 (1.58)	36.0	3.68 (1.51)	9.5	2.30 (1.39)	53.9	4.34 (1.26)
	Interpersonal	Anger-like	18.9	2.82 (1.58)	31.5	3.59 (1.50)	5.3	2.21 (1.26)	46.3	4.19 (1.23)
		Smile	40.3	4.17 (1.13)	58.9	4.52 (1.12)	31.6	3.52 (1.48)	18.0	3.06 (1.36)
		Sadness-like	18.6	3.13 (1.44)	44.7	3.95 (1.53)	10.6	2.38 (1.54)	53.0	4.25 (1.28)
	Ecstatic	Anger-like	15.2	2.84 (1.63)	27.8	3.36 (1.60)	3.1	2.15 (1.29)	50.0	4.18 (1.36)
		Smile	51.4	4.22 (1.35)	55.0	4.43 (1.18)	28.7	3.43 (1.39)	22.8	3.39 (1.36)
		Sadness-like	17.2	2.98 (1.45)	26.3	3.32 (1.42)	7.1	2.33 (1.39)	45.2	3.98 (1.39)
	Total, % agreed in at least 1 vignettes, mean (*SD*)	Anger-like	26.9	2.82 (1.22)	74.1	3.55 (0.97)	23.8	2.26 (1.00)	79.6	4.16 (1.03)
		Smile	74.4	4.14 (0.95)	87.6	4.56 (0.73)	59.1	3.56 (0.95)	45.4	3.20 (0.99)
		Sadness-like	41.6	2.90 (1.09)	61.4	3.48 (1.12)	24.1	2.34 (1.08)	79.1	4.18 (1.01)

#### Individual Difference Measure

Respondents answered a revised version of the Dimorphous Expression Questionnaire (Aragón et al., 2015). The questionnaire was modified from the original by the addition of a preface to increase participants’ understanding of what we are asking, and questions were added to reflect the greater diversity of contexts in which dimorphous expression have been reported to occur (Aragón, 2016; Aragón and Bargh, 2018). In the preface, participants viewed five photographs of men and women displaying anger and sadness expressions. On the next page the photographs had labeling that indicated the situation in which each expression had arose. Some were positive situations, and some were negative. We made clear to participants that we were interested in when normatively negative facial expressions represented positive emotions, and when normatively positive facial expressions represented negative emotions. Participants then responded to five items (α = 0.88) that captured anger expressions when feeling positive emotions, and six items (α = 0.72) that captured participants tendency to cry or appear sad when feeling positive emotions. Response options were 1 = *Strongly Disagree*, 2 = *Disagree*, 3 = *Somewhat Disagree*, 4 = *Somewhat Agree*, 5 = *Agree*, and 6 = *Strongly Agree*. See Table 2 descriptive statistics.

**TABLE 2:** Descriptive statistics for Dimorphous expression, anger and sadness expression items.

Dim.Exp	Item description	South Korea	United States	Total	*F* statistic		Percentage who agreed or strongly agreed (scores of 5 or 6)		
Mean (*SD*)	Mean (*SD*)	Mean (*SD*)	*df* = 1, 658	*p*-value	South Korea	United States	Total
Normative anger expressions	I can look angry (e.g., clenched jaw and pumping fists) when I feel intense accomplishment (for example when getting a great grade on an important exam, and shouting “YES!”).	3.55 (1.64)	4.17 (1.45)	3.88 (1.57)	26.93	<0.001	34.8	48.0	41.9
	I could make an expression that looks angry (e.g., clenched jaw and pumping fists), if I experienced a large windfall (for example winning $10 million dollar lottery).	3.54 (1.58)	3.72 (1.49)	3.64 (1.54)	2.32	0.128	31.8	34.7	33.4
	I can look angry (e.g., clenched jaw and pumping fists) when I feel intense excitement (for example when at a rock concert shouting “YEAHHH!”).	3.17 (1.57)	4.00 (1.45)	3.61 (1.56)	50.36	<0.001	23.0	42.9	33.7
	I can look angry (e.g., clenched jaw and pumping fists) when I feel intense anticipation (for example when heading into an athletic competition shouting “YEAH!”).	3.49 (1.63)	4.22 (1.33)	3.88 (1.52)	39.77	<0.001	32.5	47.7	40.7
	I can have physical expressions that might look like anger (e.g., clenched jaw, gritted teeth, pumping fists, or pinching and squeezing), when I am actually overwhelmed with positive feelings.	3.09 (1.51)	3.38 (1.40)	3.24 (1.46)	6.57	0.011	19.0	21.8	20.5

	All items	3.37 (1.30)	3.90 (1.16)	3.65 (1.25)	31.11	<0.001	% agreed to at least 1 item		
							54.4	68.1	61.8

Normative sadness expressions	I cry when I see loved ones emotionally reunite (for example when a person returns home after a long absence).	3.91 (1.11)	3.93 (1.36)	3.92 (1.25)	0.03	0.855	32.5	36.7	34.7
	I would cry if I experienced a large windfall (for example winning $10 million dollar lottery).	3.66 (1.49)	3.86 (1.51)	3.77 (1.50)	2.90	0.089	30.5	36.7	33.8
	I cry when I see a person give unselfishly to another (for example when someone donates a home to a needy family).	3.89 (1.32)	3.50 (1.27)	3.68 (1.30)	15.09	<0.001	35.7	20.9	27.8
	I cry when I achieve something that I worked long and hard to obtain (for example at graduation, or when receiving an award).	4.67 (1.23)	3.51 (1.41)	4.05 (1.45)	125.16	<0.001	63.0	25.7	42.9
	I cry when in awe of nature (for instance when looking out at a beautiful tropical island).	3.17 (1.48)	2.51 (1.29)	2.81 (1.42)	37.57	<0.001	21.6	8.5	14.6
	I cry when I feel very close to a loved one (for instance when feeling mutual love with another person).	3.73 (1.35)	3.59 (1.38)	3.65 (1.37)	1.67	0.197	31.5	26.6	28.8

	All items	3.84 (0.70)	3.48 (1.00)	3.65 (0.89)	27.30	<0.001	% agreed to at least 1 item		
							89.5	65.5	76.6

## Results

When deciding upon an analytical strategy for the experimental paradigm, we first ran a nested mixed linear model to test for effects of the counterbalance of the vignette type, counterbalance of vignette valence, the gender of model, and the background/ethnicity of the models, all of which varied on each trial. There were no significant main effects of these variables. Results were similar with and without counterbalance and model characteristic variables added as controls. We chose to run our analysis with generalized linear models because they provided effect sizes and power statistics, which are not available for nested mixed linear models. Results are in the same direction and of similar magnitude and significance with either the mixed linear or the generalized linear models. *Post hoc* comparisons have been Bonferroni corrected. We report key findings here and details in tables. Tables have been created with detailed descriptive statistics, including percentages of participants who agreed/disagreed with our prompts to provide our readers a full sense of how participants responded to our questions.

### Experimental Paradigm

#### Inferred Intensity of Emotion

**FIGURE 3:**
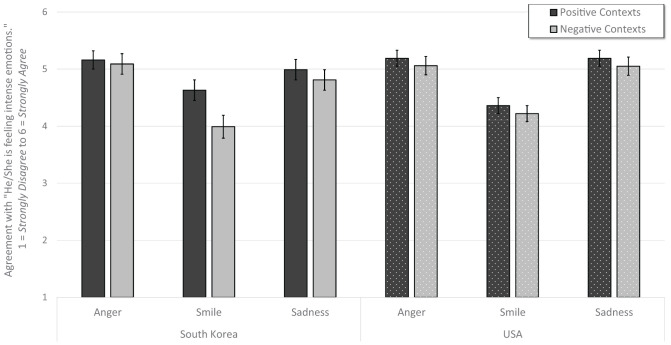
Participants who viewed anger and sadness expressions interpreted that expressers had more intense experiences than those participants who read the same 10 vignettes in the smile condition. Smiles in positive contexts were seen as more intense than in negative contexts for South Korean participants. Error bars indicate ±2 standard errors.

In a repeated measure, general linear model, we predicted intensity ratings with repeated effects of context (positive event and negative event) and vignette (5 types), with between subject effects of expression (anger, smiling, and sadness) and country (South Korea and United States). We entered all main effects and possible interactions. Consistent with past research, anger (*M* = 5.12, *SE* = 0.05) and sadness (*M* = 5.01, *SE* = 0.05) expressions communicated more intense emotional experiences than did smiles (*M* = 4.30, *SE* = 0.05), *F*(2, 569) = 74.76, *p* < 0.001, $\eta_{\text{p}}^{2}$ = 0.21, observed power = 1.00. There was also a significant country × context × expression interaction, *F*(2, 569) = 16.26, *p* < 0.001, $\eta_{\text{p}}^{2}$ = 0.02, observed power = 0.86, that revealed South Korean participants in the smile condition interpreted positive vignettes as more intense than negative vignettes. See Table 3 and Figure 3.

**TABLE 3:** General linear repeated measures models testing inferred: intensity and valence of emotion.

Description	Omnibus					Descriptive statistics								Pairwise comparisons		
	*df*	*F*	*p*-value	$\eta_{\text{p}}^{2}$	Observed power	Interaction variable	Interaction variable	Level 1	*M* (*SE*)	Level 2	*M* (*SE*)	Level 3	*M* (*SE*)	*p-*value 1 and 2	*p*-value 1 and 3	*p*-value 2 and 3
Intensity of experience analysis																
Context (pos.context, neg.context)	(1, 569)	35.65	<0.001	0.059	1.00			pos. context	4.90 (0.03)	neg. context	4.72 (0.03)	–	–	*p* < 0.001	–	–
Country (South Korea, United States)	(1, 569)	1.12	=0.291	0.002	0.18			–	–	–	–	–	–	–	–	–
Expression (anger, smile, and sadness)	(2, 569)	74.76	<0.001	0.208	1.00			Anger	5.12 (0.05)	Smile	4.30 (0.05)	Sadness	5.01 (0.05)	<0.001	=0.394	<0.001
Context × Country	(1, 569)	16.26	<0.001	0.028	0.98		*w/in pos. context*	South Korea	4.92 (0.04)	United States	4.87 (0.04)	–	–	>1.00	–	–
							*w/in neg. context*	South Korea	4.63 (0.04)	United States	4.81 (0.04)	–	–	=0.023	–	–
Context × Expression	(2, 569)	15.96	<0.001	0.053	1.00		*w/in pos. context*	Anger	5.11 (0.06)	Smile	4.49 (0.06)	Sadness	5.09 (0.06)	<0.001	>1.00	<0.001
							*w/in neg. context*	Anger	5.13 (0.06)	Smile	4.10 (0.06)	Sadness	4.93 (0.06)	<0.001	=0.053	<0.001
Country × Expression	(2, 569)	8.18	=0.184	0.006	0.36			–	–	–	–	–	–	–	–	–
Context × Country × Expression	(2, 569)	5.63	=0.004	0.019	0.859	*w/in South Korea*	*w/in pos. context*	Anger	5.16 (0.08)	Smile	4.63 (0.09)	Sadness	4.99 (0.09)	<0.001	=0.462	=0.012
							*w/in neg. context*	Anger	5.09 (0.09)	Smile	3.99 (0.10)	Sadness	4.81 (0.09)	<0.001	=0.078	<0.001
						*w/in United States*	*w/in pos. context*	Anger	5.19 (0.07)	Smile	4.36 (0.07)	Sadness	5.19 (0.07)	<0.001	=0.687	<0.001
							*w/in neg. context*	Anger	5.16 (0.08)	Smile	4.22 (0.07)	Sadness	5.05 (0.08)	<0.001	=0.867	<0.001
Affect valence analysis																
Context (pos. context, neg. context)	(1, 578)	84.80	<0.001	0.128	1.00			pos. context	3.47 (0.01)	neg. context	3.43 (0.01)	–	–	*p* < 0.001	–	–
Country (South Korea, United States)	(1, 578)	28.74	<0.001	0.047	1.00			South Korea	3.35 (0.02)	United States	3.46 (0.01)	–	–	*p* < 0.001	–	–
Expression (anger, smile, and sadness)	(2, 578)	1.54	=0.214	0.005	0.33			–	–	–	–	–	–	–	–	–
Valence (pos.emotion, neg.emotion)	(1, 578)	0.57	=0.450	0.001	0.12			–	–	–	–	–	–	–	–	–
Context × Valence	(1, 578)	1508.47	<0.001	0.723	1.00		*w/in pos. context*	pos.emo.	4.61 (0.04)	neg.emo.	2.17 (0.04)	–	–	<0.001	–	–
							*w/in neg. context*	pos.emo.	2.33 (0.04)	neg.emo.	4.52 (0.04)	–	–	<0.001	–	–
Expression × Valence	(2, 578)	151.10	<0.001	0.343	1.00		*w/in anger*	pos.emo.	3.06 (0.04)	neg.emo.	3.80 (0.04)	–	–	<0.001	–	–
							*w/in smile*	pos.emo.	3.89 (0.04)	neg.emo.	2.88 (0.04)	–	–	<0.001	–	–
							*w/in sadness*	pos.emo.	3.23 (0.04)	neg.emo.	3.59 (0.04)	–	–	<0.001		
Country × Valence	(1, 578)	0.410	=0.522	0.001	0.01			–	–	–	–	–	–	–	–	–
Context × Expression × Valence	(2, 578)	8.94	<0.001	0.030	0.97	*w/in pos. context*	*w/in pos.emo*	Anger	4.25 (0.07)	Smile	4.99 (0.07)	Sadness	4.60 (0.07)	<0.001	=0.001	=0.001
							*w/in neg.emo*	Anger	2.68 (0.07)	Smile	1.98 (0.07)	Sadness	2.33 (0.07)	<0.001	=0.001	<0.001
						*w/in neg. context*	*w/in pos.emo*	Anger	1.87 (0.06)	Smile	2.79 (0.07)	Sadness	1.86 (0.06)	<0.001	*>* 1.00	<0.001
							*w/in neg.emo*	Anger	4.92 (0.06)	Smile	3.79 (0.06)	Sadness	4.84 (0.06)	<0.001	*>* 1.00	<0.001
Context × Country × Valence	(1, 578)	6.19	=0.013	0.011	0.70	*w/in pos. context*	*w/in pos.emo*	South Korea	4.52 (0.06)	United States	4.71 (0.05)	–	–	=0.020	–	–
							*w/in neg.emo*	South Korea	2.36 (0.06)	United States	2.31 (0.05)	–	–	=0.545	–	–
						*w/in neg. context*	*w/in pos.emo*	South Korea	2.19 (0.06)	United States	2.16 (0.05)	–	–	=0.690	–	–
							*w/in neg.emo*	South Korea	4.36 (0.06)	United States	4.68 (0.05)	–	–	<0.001	–	–
Expression × Valence × Country	(2, 578)	1.16	=0.315	0.004	0.25			–	–	–	–	–	–	–	–	–
Country	(2, 578)	0.452	=0.637	0.002	0.12			–	–	–	–	–	–	–	–	–

**All post hoc pairwise comparison p-values have been Bonferroni corrected for multiple comparisons*.*

#### Inferred Emotional Experience

**FIGURE 4:**
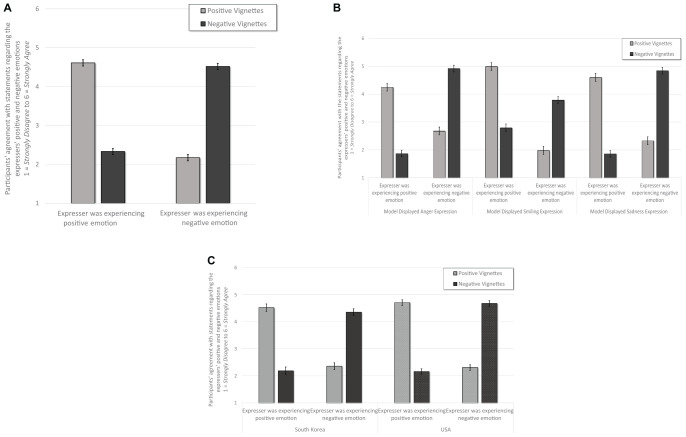
Overall, positive contexts were deemed to produce positive- not negative emotions, and negative contexts were deemed to produce negative – not positive emotions, as in every case means and deviations clearly fell within agreement and disagreement categories. Panel **(A)** illustrates that participants’ inferences about the valence of experience for the expresser was chiefly determined by the positive or negative framing of the context. Panel **(B)** shows a moderation by expression, which was chiefly driven by smiles seen as representing lower negative and higher positive emotions in negative situations. Panel **(C)** illustrates that American participants rated higher positive emotion in positive vignettes, and higher negative emotion in negative vignettes than did South Korean participants. Error bars indicate ±2 standard errors.

In a repeated measure, general linear model, we predicted affective valence with repeated effects of context (positive event and negative event), vignette (5 types), and valence (positive and negative), with between subject effects of expression (anger, smiling, and sadness) and country (South Korea and United States). We entered all main effects and possible interactions. One interaction accounted for the majority of the variance explained by the model, that was the interaction between context and valence, *F*(1, 578) = 1508.47, *p* < 0.001, $\eta_{\text{p}}^{2}$ = 0.72, observed power = 1.00 (see Figure 4A). Emotions in positive contexts were interpreted as predominantly positive (*M* = 4.61, *SE* = 0.04), not negative (*M* = 2.33, *SE* = 0.04). In contrast, emotions in negative contexts were interpreted as predominantly negative (*M* = 4.52, *SE* = 0.04), not positive (*M* = 2.17, *SE* = 0.04).

A far less robust, yet significant interaction was context × valence × country, *F*(1, 578) = 6.19, *p* = 0.013, $\eta_{\text{p}}^{2}$ = 0.01, observed power = 0.70 (see Figure 4B). American participants (*M* = 4.71, *SE* = 0.05) interpreted more positivity in positive contexts than did South Korean participants (*M* = 4.52, *SE* = 0.06), *p* = 0.020, and American participants (*M* = 4.68, *SE* = 0.05) interpreted more negativity in negative contexts than did South Korean participants (*M* = 4.36, *SE* = 0.06), *p* < 0.001. The interaction between context and valence was also moderated by expression, *F*(2, 578) = 8.94, *p* < 0.001, $\eta_{\text{p}}^{2}$ = 0.03, observed power = 0.97. This moderation was mainly driven by the smile condition. Smiles featured in negative vignettes were interpreted as representing less negative emotion (*M*_neg._ = 3.79, *SE* = 0.06) than were anger (*M*_neg._ = 4.92, *SE* = 0.06) and sadness (*M*_neg._ = 4.84, *SE* = 0.06) expressions. See Table 3 and Figure 4C.

#### Inferred Motivational Orientations

**FIGURE 5:**
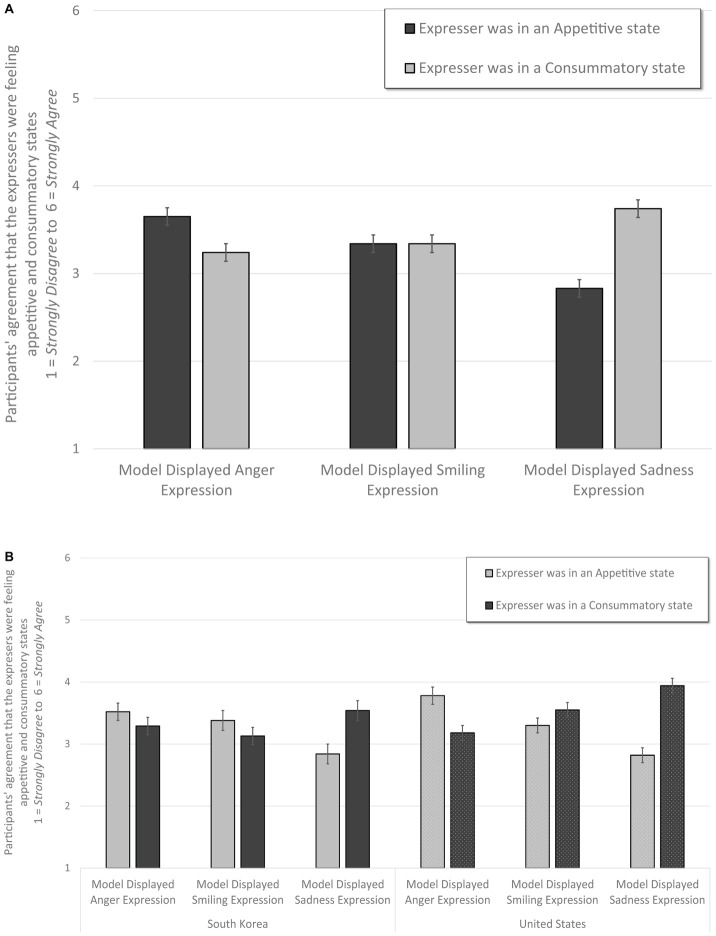
Panel **(A)** illustrates that across these five different “flavors” of emotion, and both positive and negative events, anger expressions communicated more appetitive, less consummatory experiences, and sadness expressions communicated more consummatory, less appetitive experiences. Smiles did not differentiate between these two motivational aspects. Panel **(B)** illustrates that this largely held true cross culturally, but South Korean participants rated lower appetitive for anger and lower consummatory for sadness and smile expressions than did American participants. Error bars indicate ±standard errors.

Using the same statistical strategy, we tested participants’ inferences about our models’ appetitive and consummatory motivations. See Table 4. Central to this investigation and as hypothesized, there was a significant interaction between expression and motivational orientation, *F*(2, 578) = 86.14, *p* < 0.001, $\eta_{\text{p}}^{2}$ = 0.23, observed power = 1.00. When participants were assigned randomly to view anger expressions, they interpreted that the expressers had higher appetitive (*M* = 3.65, *SE* = 0.05) than consummatory (*M* = 3.24, *SE* = 0.05) orientations, *p* < 0.001. In contrast, those participants assigned randomly to view sadness expressions inferred higher consummatory (*M* = 3.74, *SE* = 0.05) than appetitive (*M* = 2.83, *SE* = 0.05) orientations, *p* < 0.001. Participants who viewed smiles, did not distinguish between appetitive (*M* = 3.34, *SE* = 0.05) or consummatory motivations (*M* = 3.34, *SE* = 0.05), *p* = 1.00. Replicating past work (Aragón and Bargh, 2018), these effects did not depend upon whether the expressions arose in positive or negative contexts, as the expression × motivation × context interaction was not significant, *F*(2, 578) = 0.139, *p* = 0.870. See Figure 5A.

**TABLE 4:** General linear repeated measures models testing inferred motivational orientations, and seen, known, used items.

Description	Omnibus					Descriptive statistics								Pairwise comparisons		
	*df*	*F*	*p*-value	$\eta_{\text{p}}^{2}$	Observed power	Interaction variable	Interaction variable	Level 1	*M* (*SE*)	Level 2	*M* (*SE*)	Level 3	*M* (*SE*)	Levels 1 and 2	Levels 1 and 3	Levels 2 and 3
Motivational orientations analysis																
Context (pos. context, neg. context)	(1, 578)	42.77	<0.001	0.069	1.00			pos. context	3.43 (0.02)	neg. context	3.28 (0.02)	–	–	<0.001	–	–
Country	(1, 578)	14.85	<0.001	0.025	0.970			South Korea	3.28 (0.03)	United States	3.43 (0.02)	–	–	<0.001	–	–
Expression	(2, 578)	6.42	=0.002	0.022	0.903			Anger	3.44 (0.03)	Smile	3.34	Sadness	3.28 (0.03)	<0.001	=0.635	=0.077
Motivation (appetitive., consumm.)	(1, 578)	15.03	<0.001	0.025	0.972			Appetitive.	3.27 (0.03)	consumm.	3.44 (0.03)	–	–	<0.001	–	–
Context × Motivation	(1, 578)	211.92	<0.001	0.268	1.00		*pos. context*	Appetitive.	3.64 (0.04)	consumm.	3.21 (0.04)	–	–	<0.001	–	–
							*neg. context*	Appetitive.	2.90 (0.04)	consumm.	3.67 (0.04)	–	–	<0.001	–	–
Expression × Motivation	(2, 578)	86.14	<0.001	0.230	1.00		*w/in anger*	Appetitive.	3.65 (0.05)	consumm.	3.24 (0.05)	–	–	<0.001	–	–
							*w/in smile*	Appetitive.	3.34 (0.05)	consumm.	3.34 (0.05)	–	–	=0.997	–	–
							*w/in sadness*	Appetitive.	2.83 (0.05)	consumm.	3.74 (0.05)	–	–	<0.001	–	–
Country × Motivation	(1, 578)	4.614	=0.032	0.008	0.573	*w/in appetitive.*		South Korea	3.25 (0.04)	United States	3.30 (0.04)	–	–	=0.352	–	–
						*w/in consumm.*		South Korea	3.32 (0.04)	United States	3.56 (0.04)	–	–	<0.001	–	–
Context × Expression × Motivation	(2, 578)	0.139	=0.870	0.000	0.07			–	–	–	–	–	–	–	–	–
Context × Country × Motivation	(1, 578)	341.07	<0.001	0.371	1.00	*w/in pos. context*	*w/in app.mot.*	South Korea	3.95 (0.06)	United States	3.34 (0.05)	–	–	<0.001	–	–
							*w/in cons.mot.*	South Korea	2.66 (0.05)	United States	3.75 (0.05)	–	–	<0.001	–	–
						*w/in neg. context*	*w/in app.mot.*	South Korea	2.55 (0.06)	United States	3.26 (0.05)	–	–	<0.001	–	–
							*w/in cons.mot.*	South Korea	3.98 (0.06)	United States	3.36 (0.05)	–	–	<0.001	–	–
Expression × Motivation × Country	(2, 578)	10.76	0.000	0.036	0.99	*w/in anger*	*w/in app.mot.*	South Korea	3.52 (0.07)	United States	3.78 (0.07)	–	–	=0.009	–	–
							*w/in cons.mot.*	South Korea	3.29 (0.07)	United States	3.18 (0.06)	–	–	=0.244	–	–
						*w/in smile*	*w/in app.mot.*	South Korea	3.38 (0.08)	United States	3.30 (0.06)	–	–	=0.478	–	–
							*w/in cons.mot.*	South Korea	3.13 (0.08)	United States	3.55 (0.06)	–	–	<0.001	–	–
						*w/in sadness*	*w/in app.mot.*	South Korea	2.84 (0.08)	United States	2.82 (0.06)	–	–	=0.802	–	–
							*w/in cons.mot.*	South Korea	3.54 (0.07)	United States	3.94 (0.06)	–	–	<0.001	–	–
Context × Exp. × Motiv. × Country	(2, 578)	0.686	=0.504	0.002	0.17			–	–	–	–	–	–	–	–	–
Seen, known, and used the expressions analysis																
Context (pos. context, neg. context)	(1, 566)	101.41	<0.001	0.152	1.00			pos. context	3.94 (0.04)	neg. context	3.53 (0.04)	–	–	<0.001	–	–
Country (South Korea, United States)	(1, 566)	178.16	<0.001	0.239	1.00			South Korea	3.30 (0.05)	United States	4.17 (0.04)	–	–	<0.001	–	–
Expression (anger, sadness, and smile)	(2, 566)	23.64	<0.001	0.077	1.00			Anger	3.52 (0.06)	Smile	4.05 (0.06)	Sadness	3.63 (0.06)	<0.001	=0.173	<0.001
Item (seen, known, and used)	(2, 566)	293.75	<0.001	0.342	1.00			Seen	3.92 (0.04)	Known	3.86 (0.03)	Used	3.42 (0.04)	=0.001	<0.001	<0.001
Context × Country	(1, 566)	58.53	<0.001	0.094	1.00	*w/in pos. context*		South Korea	3.65 (0.06)	United States	4.22 (0.05)	–	–	<0.001	–	–
						*w/in neg. context*		South Korea	2.94 (0.06)	United States	4.12 (0.05)	–	–	<0.001	–	–
Context × Expression	(2, 566)	50.10	<0.001	0.150	1.00	*w/in anger*		pos. context	3.55 (0.07)	neg. context	3.50 (0.07)	–	–	=0.446	–	–
						*w/in smile*		pos. context	4.53 (0.07)	neg. context	3.56 (0.07)	–	–	<0.001	–	–
						*w/in sadness*		pos. context	3.73 (0.07)	neg. context	3.54 (0.07)	–	–	=0.006	–	–
Country × Expression	(2, 566)	37.80	<0.001	0.118	1.00	*w/in anger*		South Korea	2.91 (0.08)	United States	4.13 (0.08)	–	–	<0.001	–	–
						*w/in smile*		South Korea	4.01 (0.09)	United States	4.08 (0.07)	–	–	=0.568	–	–
						*w/in sadness*		South Korea	2.96 (0.09)	United States	4.30 (0.07)	–	–	<0.001	–	–
Context × Expression × Country	(2, 566)	57.32	<0.001	0.168	1.00	*w/in pos. context*	*w/in anger*	South Korea	3.24 (0.10)	United States	3.85 (0.09)	–	–	<0.001	–	–
							*w/in smile*	South Korea	4.35 (0.11)	United States	4.72 (0.08)	–	–	=0.006	–	–
							*w/in sadness*	South Korea	3.37 (0.10)	United States	4.08 (0.08)	–	–	<0.001	–	–
						*w/in neg. context*	*w/in anger*	South Korea	2.58 (0.10)	United States	4.41 (0.09)	–	–	<0.001	–	–
							*w/in smile*	South Korea	3.68 (0.11)	United States	3.44 (0.08)	–	–	=0.079	–	–
							*w/in sadness*	South Korea	2.56 (0.10)	United States	4.51 (0.09)	–	–	<0.001	–	–

**All post hoc pairwise comparison p-values have been Bonferroni corrected for multiple comparisons*.*

There was also a less robust yet significant interaction between expression × motivation × country *F*(2, 578) = 10.76, *p* < 0.001, $\eta_{\text{p}}^{2}$ = 0.04, observed power = 0.99. American participants (*M* = 3.78, *SE* = 0.07) reported slightly higher agreement that anger expressions were appetitive than did South Korean participants (*M* = 3.52, *SE* = 0.07), *p* = 0.009. Americans (*M* = 3.94, *SE* = 0.06) reported slightly higher agreement that sadness expressions were consummatory than did South Korean participants (*M* = 3.54, *SE* = 0.07), *p* < 0.001. And South Korean participants (*M* = 3.13, *SE* = 0.08) did infer that smiles were less consummatory than did American participants (*M* = 3.55, *SE* = 0.06), *p* < 0.001. There was no significant interaction between context × expression × motivation × country, *p* = 0.504. See Figure 5B.

#### Seen, Known, Used: Anger, Sadness, and Smiling Expressions

Data were again analyzed in a repeated measure, general linear model, with repeated effects of item (seen, known, and used) × valence (positive and negative), and vignette (5 types). Condition (anger, smiling, and sadness) and country were entered as fixed factors. As one would expect, participants reported strongest agreement for having seen an expression (*M* = 3.92, *SE* = 0.04), next highest for having known someone who expresses in such a manor (*M* = 3.86, *SE* = 0.03), and lowest scores for having expressed in such a way themselves (*M* = 3.42, *SE* = 0.04), *F*(1,566) = 293.75, *p* < 0.001, $\eta_{\text{p}}^{2}$ = 0.34, observed power = 1.00, all pairwise *p*’s < 0.001. Item and vignette type did not interact with country, valence and country, or valence, expression and country, all *p*’s > 0.05. Results indicated that all three questions showed a consistency that occurred across all vignette types and will be reported out here as prevalence of these expressions. Vignette and item-specific details are offered in Table 1.

**FIGURE 6:**
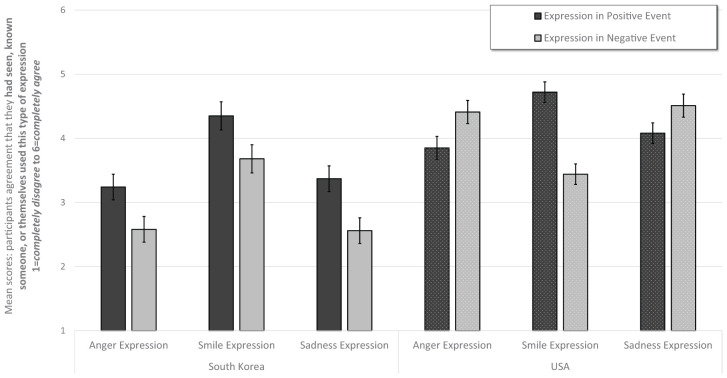
Figure illustrates the prevalence of anger, sadness or smiling expression in positive and negative events. Error bars indicate ±2 standard errors.

As hypothesized, there was a large main effect of country *F*(1,566) = 178.16, *p* < 0.001, $\eta_{\text{p}}^{2}$ = 0.24, observed power = 1.00, with South Korean participants reporting a lower prevalence of the depicted expressions (*M* = 3.30, *SE* = 0.05) than American participants (*M* = 4.17, *SE* = 0.04). There was a robust interaction between valence × expression × country, *F*(2,566) = 57.32, *p* < 0.001, $\eta_{\text{p}}^{2}$ = 0.17, observed power = 1.00. American participants reported a higher prevalence of anger and sadness expressions in negative contexts (*M*_ang._ = 4.41, *SE* = 0.09; *M*_sad._ = 4.51, *SE* = 0.09) than in positive contexts (*M*_ang._ = 3.85, *SE* = 0.09; *M*_sad._ = 4.08, *SE* = 0.08). In contrast, South Korean participants reported a higher prevalence of anger and sadness expressions in positive contexts (*M*_ang._ = 3.24, *SE* = 0.10; *M*_sad._ = 3.37, *SE* = 0.10) than in negative contexts (*M*_ang._ = 2.58, *SE* = 0.10; *M*_sad._ = 2.56, *SE* = 0.10) contexts, all pairwise *p*’s < 0.001. Additionally, although South Korean and American participants reported higher prevalence of smiles in positive contexts (*M*_SK_ = 4.35, *SE* = 0.11; *M*_USA_ = 4.72, *SE* = 0.10) than in negative contexts (*M*_SK_ = 3.68, *SE* = 0.11; *M*_USA_ = 3.44, *SE* = 0.08), both *p*’s < 0.00, American participants reported a higher prevalence of smiles in positive contexts than did South Korean participants, *p* = 0.006. South Korean participants reported marginally higher prevalence of smiles in negative contexts than did American participants, *p* = 0.079. See Table 4 and Figure 6.

#### Individual Difference Measure of Positive Emotions Expressed Through Anger and Sadness

**FIGURE 7:**
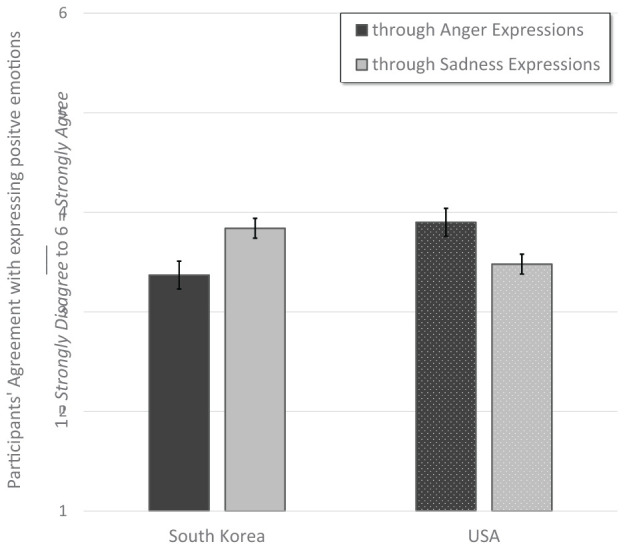
Panel illustrates that South Korean participants reported expressing positive emotions with more sadness and fewer anger expressions than did American participants. Error bars indicate ±2 standard errors.

The same analytic strategy was used to test the prevalence of positive emotion expressed through anger and sadness displays. Overall, South Korean participants (*M* = 3.60, *SE* = 0.05) did not differ from American participants (*M* = 3.69, *SE* = 0.04) in their reports of displaying normatively negative expressions when feeling highly positive emotions. There was a significant interaction between country and type of expression (anger and sadness), *F*(1, 567) = 61.17, *p* < 0.001, $\eta_{\text{p}}^{2}$ = 0.09, observed power = 1.00. South Korean participants (*M* = 3.84, *SE* = 0.05) reported higher usage of sadness expressions within positive contexts than did American participants (*M* = 3.48, *SE* = 0.05), *p* < 0.001. In contrast, American participants (*M* = 3.90, *SE* = 0.07) reported a higher usage of anger expressions in positive situations than did South Korean participants (*M* = 3.37, *SE* = 0.07), *p* < 0.001. See Figure 7.

In the dimorphous expression questionnaire there was an interaction of higher use of sadness than anger expressions by South Korean participants, and the higher use of anger than sadness expressions by American participants. In the item that asked participants if they had used expressions, in the mean scores the interaction was not apparent. However, when we created binary scores (those who agreed or strongly agreed that they had expressed) for our descriptive statistics, the interaction was again evident, in that for our South Korean participants 41.6% agreed that they had used the sadness expression and 26.9% agreed to have used the anger expression in at least one of the positive vignettes. In contrast, in our American participants 74.1% agreed that they had used the anger expressions, and 61.4% agreed to have used the sadness expressions in at least one of the positive vignettes.

## Discussion

Our central question pertained to the existence of dimorphous expressions across these two cultures in South Korea and the United States. It appears that dimorphous expressions do exist in South Korea as they have been observed in the United States, in that there are instances in which individuals report to use normatively negative expressions to express positive emotions. As well, participants from South Korea generally interpreted anger and sadness expressions within positive contexts as representing predominantly positive- not negative, or positive and negative mixed or sequentially experienced emotions. Also, consistent with dimorphous theory, anger and sadness expressions situated within positive contexts were interpreted as representing intense emotional experiences. When participants were queried if they had seen, known, or themselves used anger and sadness expressions within positive contexts, again, there was evidence for the existence of dimorphous expressions in both cultures.

An interesting pattern emerged when participants reported on their own use of anger and sadness displays when feeling positive emotions. When asked through the dimorphous expression questionnaire, the overall prevalence of dimorphous expressions did not differ by country. However, when asked within specific vignettes, and provided with specific exemplars of expression, there was a large main effect of country with South Korean participants reporting overall a lower agreement in using anger and sadness displays within positive contexts (we discuss negative contexts below). It could very well be that the vignettes were not equally compatible for both samples, i.e., if the specific vignettes did not tap into South Korean experiences as well as they had American, we might have inadvertently created the main effect of culture. Differences between South Korea and the United States did not appear for the dimorphous expression questionnaire, and those questions asked more generally about expression with a greater breadth of instances in which dimorphous expressions occur. This investigation provided evidence that dimorphous expressions exist in both South Korea and the United States, and future research will be needed to determine the extent to which these dimorphous expressions are used.

In regard to the communication of motivational orientations through expressions, the experimental portion conceptually replicated Aragón and Bargh (2018). Anger and sadness expressions communicated appetitive and consummatory motivations, respectively, in both positive and negative contexts in both the United States and South Korea. Consistent with past research, smiling expressions did not provide consistent signals about appetitive or consummatory orientations. This pattern of results speaks to a possible functional reason for why smiles are not the only expressions that arise for positive feelings. When experiencing highly intense positive feelings paired with an antsy feeling of wanting to go or a consuming feeling of wanting to stop, anger and sadness displays, respectively, communicate those feelings better than do smiles. It seems that this would be important social information to be able to communicate when emotions are running high for the coordination, cooperation, and compensatory behaviors that facilitate social interactions.

Another intriguing pattern in our results was that in many of the analyses South Korean and American participants were in agreement as to what a certain expression did not represent, but when it came to stating what the expression did represent it seemed that the South Korean participants were less adamant about what they were viewing. For example, both South Korean and American participants agreed that anger and sadness expressions in negative contexts were not representing positive emotions, but South Koreans appeared less adamant that they were negative emotions. The same was true for anger and sadness expressions in positive contexts, there was cross-cultural agreement that they were not negative experiences, but South Korean participants were less extreme in rating how positive they were. The same pattern emerged when evaluating anger and sadness expressions for motivational orientations. There was cross-cultural agreement that anger expressions were not consummatory and sadness expressions were not appetitive, but again, South Korean participants were not as emphatic that anger expressions were appetitive and sadness expressions were consummatory as American participants. This suggests a possible reporting bias because in each case, South Korean participants were on par with Americans in declaring what an expression did not communicate, but they were less confident to say what it did communicate.

The addition of the smiling condition was intended as type of control condition because smiles are presumed to be the normative expression for positive emotions, and our central aim was to understand anger and sadness displays in positive contexts in these two cultures. However, the smile condition provided the most complex, least straightforward results of our investigation. Here we attempt to address these complexities. Concerning what was communicated by smiles, generally, when smiles were presented in positive contexts they were interpreted as representing positive experiences, and when smiles were presented in negative contexts they were interpreted as representing negative experiences. As previous research has demonstrated smiles did not clearly communicate appetitive or consummatory motivations, but South Korean participants appeared to read smiles as being more so appetitive and less consummatory than did American participants (see Figure 5B). Work in Affect Valuation Theory has found that more subtle smiles are more so the norm in Eastern relative to Western contexts (Tsai et al., 2019). The types of smiles in our stimuli contained 7 smiles that exposed teeth, and 4 which were open-mouthed. Thus, it is possible that the larger, toothier grins may have seemed to communicate a higher-arousal emotion to our South Korean participants.

As previous research had suggested South Korean participants did endorse that they had seen, known and used smiles more so in negative contexts than did American participants. This phenomenon is thought to represent masking of negative emotions. We note though that smiles in negative contexts could represent masking, but they could also represent dimorphous expressions of negative emotions, reappraisal of the negative experience, or mixed or sequentially experienced positive and negative emotions. In a similar experiment that provided participants text boxed with which to comment about how they would feel if smiling in context of losing an important sporting event (Aragón, 2020), participants noted masking “(I would) try to not show how I am having very negative feelings about losing,” dimorphous expressions “I would feel so frustrated and upset that I would laugh. Sometimes when I am frustrated or fed up with something, I laugh (but not because I am happy),” reappraisal “I was pleased with my effort and gave it everything I had,” and mixed or sequentially experienced positive and negative emotions “I would feel discouraged but also proud.” Therefore, it is possible that any of these factors, i.e., masking, dimorphous expression of negative emotions, the tendency to reappraise (Butler et al., 2007; English et al., 2017), or the prevalence of mixed or sequential emotions could account for the differences we observed in the interpretation and prevalence of smiles across both positive and negative contexts in South Korean and American participants. These findings will be interesting to probe in future research.

Additionally, previously reported display rules research has shown that individuals from eastern contexts are less likely to express negative emotions than are those from western contexts. However, the results from the self-report dimorphous expression questionnaire suggest that those display rules may be less tied to physical displays and more tied to rules about which emotional experiences are appropriate to communicate, because overall normatively negative displays that communicated positive emotional experiences were equally prevalent in both American and South Korean participants. In the items that asked if participants had seen, known or used expressions, expression and emotion appeared to contribute independently to the prevalence of such expressions, because anger and sadness displays were less prevalent in South Korean participants (effect of expression), but particularly so when they were communicating negative emotions (moderated by the effect of the communicated valence of emotion).

Most astounding was the interaction between country, context, and expression in regard to anger and sadness displays in the items that asked participants about having seen, known, or used expressions in the given situations. South Korean participants reported a lower prevalence in the use of anger and sadness expressions overall, but if they were to use those expressions, they reported using those expressions to express positive more so than negative emotions. The reverse was true for our American participants, who were significantly more likely to use anger and sadness expressions for the display of negative than positive emotions. Future work might explore if people from Eastern contexts might try to maintain social harmony through the use of dimorphous expressions, particularly the sadness expression. In an Eastern context that is sensitive to power distance, particularly in cases in which one is opened up to envious attacks such as when an individual has won an award, or experienced a great windfall, it might be prudent to express positive emotions through sadness or crying because such displays been found to reduce aggressive sentiments and upregulate caring responses toward the expresser (Hendriks et al., 2008; Aragón and Clark, 2018).

Limitations of this investigation include those issues highlighted above, i.e., the use of more pronounced smiles and the use of vignettes that might not have been equally compatible for both cultures. This study is also limited in that it was entirely self-report, and as such is vulnerable to issues of self-knowledge and self-presentation. Likewise, the study was conducted online, which always leaves open the possibility waning attention and effort provided by the study’s participants. Another limitation is that our stimuli is not equivalent to real-life instances. We attempted to ameliorate this shortcoming by using many different types of exemplars of expression, with both male and female models, across different types of scenarios.

Returning to the idea presented in the opening of this article, the assumption of a 1:1 correspondence between expressive displays and discrete emotional experiences would have precluded this investigation. Participants in this study and others have demonstrated and reported that expressions normatively considered negative, in this case anger and sadness expressions, can represent and communicate either positive or negative states, and can appear in different types of contexts that should elicit different what we call “flavors” of emotion. This investigation found the first evidence in both South Korea and the United States that anger and sadness displays communicate appetitive and consummatory motivational orientations, respectively. It *could* be that anger and sadness displays simply are expressions of intensity and motivation. Of course, future work will need to be done to know if that might be true. Clearly future investigations that explore manipulations of in-group/out-group status, gender, or social status of the expresser could prove interesting. Also, future research might consider if a singular expresser versus multiple expressers interact with culture. An overarching conclusion is that sometimes questioning the foundation on which our work in non-verbal behavior has been built can lead to questions that may inform subsequent work in ways that had not been considered. We hope to have made such a contribution with this work.

## Data Availability Statement

The raw data supporting the conclusions of this article will be made available by the authors, without undue reservation.

## Ethics Statement

The studies involving human participants were reviewed and approved by Clemson IRB. The patients/participants provided their written informed consent to participate in this study.

## Author Contributions

SS and OA designed and implemented the experiments, with input from AC. OA analyzed the data. SS, AC, and OA wrote the manuscript. All authors contributed to the article and approved the submitted version.

## Conflict of Interest

The authors declare that the research was conducted in the absence of any commercial or financial relationships that could be construed as a potential conflict of interest.
