# Supplementary material for: Anger and Sadness Expressions Situated in Both Positive and Negative Contexts: An Investigation in South Korea and the United States
Source: Front Psychol. 2021 Jan 13;11:579509. doi: 10.3389/fpsyg.2020.579509 (PMC7838562; doi:10.3389/fpsyg.2020.579509)
Supplement: Supplementary file 1 [file Data_Sheet_1.pdf]

Appendix A

Details of the full survey. Sections 2, 5, and 7 are reported in this manuscript.

Section	Topic	Description	Number of Questions
1	Consent	Participants were informed the purpose of research and asked for consent to participate in the survey.	1
2	Dimorphous Expression Questionnaire (Aragón et al., 2015)	This section asked participants about their own use of dimorphous expressions. Four questions (α = .80) captured expressions that look like pain when experiencing great pleasure, when thoroughly amused, when overwhelmed from a situation such as meeting a favorite celebrity, and a general item about appearing pained when overwhelmed with positive feelings. Three questions (α = .82) captured expressions that look liked disgust when experiencing great pleasure, when thinking that something is impressive, and when thinking that something is really good. Dimorphous expressions of negative emotion were also captured with seven questions about smiling and seven questions about laughing when angry, frustrated, sad, anxious, disgusted, embarrassed, and hopeless. Eight additional items captured dimorphous expressions toward cute beings (babies or animals). These items are not within the scope of this paper but available upon request to the authors.	40
3	Berkeley Expressivity Questionnaire (Gross & John, 1995)	This section asked participants about their own expressivity considering strength of emotion, and dysregulation, as modified in Aragon et al., 2015.	32
4	Likelihood Experiment	The purpose of this section was to understand cross-cultural differences in the interpretation of facial expressions when context clues are not provided, including manipulations of the expresser as ingroup or outgroup.	30
5	Vignettes: Experimental Portion	The purpose of this section was to understand what anger, smiling, and sadness displays communicated cross-culturally.	120
6	Consumers' preference	The purpose of this section was to understand alignments between expressions and products. Participants saw different facial expressions that were reactions to movie trailers, and then asked about their preferences for the advertised films.	15
7	Affect Valuation (Tsai et al., 2006)	This measure asked participants about their ideal and typically experienced affect.	60
8	Responses to cute stimuli, (Aragón et al., 2015)	This section replicated Aragon et. al., 2015, which measured participants' aggressive-type responses to cute or less cute stimuli.	18
9	Demographics	Participants were asked demographics, if they were raised in South Korea (or USA), their university, and if they were read children's books when younger.	9

Appendix B

In this table are the vignettes presented to participants. Each participant viewed both positive and negative versions of each type of vignette, counterbalanced in order of valence, with all positive and all negative vignettes presented consecutively.

	Positive Vignettes		Negative Vignettes		Positive Vignettes		Negative Vignettes	
Scenario	*Korean version*	*English version*	*Korean version*	*English version*	Considered Positive*	Considered Negative*	Considered Positive*	Considered Negative*
Windfall, Lifelong Dreams Come True	이번 상황은 한 남성(여성)이 평생의 꿈을 이룰 기회를 가졌을 때를 상상해 주시기 바랍니다.<br><br>남성(여성)은 그 사실을 알았을 때, 강렬한 감정을 느꼈습니다. 다음 사진은 좋은 소식을 들었던 순간 남성(여성)의 반응입니다.	In this scenario, we would like you to imagine that a man (woman) was given a chance to fulfill a lifelong dream.<br><br>When he (she) found out, the man felt strong emotions. This is how he (she) reacted in the moment when he (she) heard the good news.	이번 상황은 한 남성(여성)이 평생의 꿈을 이룰 기회를 놓쳤을 때를 상상해 주시기 바랍니다.<br><br>남성(여성)은 그 사실을 알았을 때, 강렬한 감정을 느꼈습니다. 다음 사진은 나쁜 소식을 들었던 순간 남성(여성)의 반응입니다.	In this scenario, we would like you to imagine that a man (woman) has lost a chance to fulfill a lifelong dream.<br><br>When he (she) found out, the man (woman) felt strong emotions. This is how he (she) reacted in the moment when he (she) heard the bad news.	*M* = 4.41<br>*SE* = .19	*M* = 1.55<br>*SE* = .20	*M* = 2.11<br>*SE* = .21	*M* = 3.85<br>*SE* = .23
See Beautiful Nature	이번 상황은 한 남성(여성)이 본인이 매우 중요하게 여기는 아름다운 자연경관을 바라보는 순간을 상상해주시기 바랍니다.<br><br>남성(여성)은 그 아름다운 경관을 보며 강렬한 감정에 빠졌습니다. 다음 사진은 아름다운 자연경관을 본 순간 남성(여성)의 반응입니다.	In this scenario, we would like you to imagine a man (woman) looking out at a beautiful nature scene that is very important to him (her).<br><br>The beauty overwhelmed him (her) with strong emotions. This is how he (she) reacted in the moment when he (she) saw the beautiful nature scene.	이번 상황은 한 남성(여성)이 본인이 매우 중요하게 여기는 아름다운 자연경관을 바라볼 기회를 놓쳤을 때를 상상해 주시기 바랍니다.<br><br>남성(여성)은 강렬한 감정에 빠졌습니다. 다음 사진은 아름다운 자연경관을 볼 기회를 놓친 순간 남성(여성)의 반응입니다.	In this scenario, we would like you to imagine a man (woman) who missed out on seeing a beautiful nature scene that is very important to him(her).<br><br>He (She) was overwhelmed with strong emotions. This is how he (she) reacted in the moment when he (she) missed out on seeing the beautiful nature scene.	*M* = 4.24<br>*SE* = .21	*M* = 1.56<br>*SE* = .20	*M* = 2.22<br>*SE* = .23	*M* = 3.37<br>*SE* = .22
Earned, Achieve Long-Term Goals	이번 상황은 한 남성(여성)이 성공하기 위해 오랜 시간 노력하여 목표달성한 순간을 상상해 주시기 바랍니다.<br><br>남성(여성)은 목표달성을 했을 때, 강렬한 감정에 빠졌습니다. 다음 사진은 목표달성에 성공한 순간 남성(여성)의 반응입니다.	In this scenario, we would like you to imagine a man (woman) who accomplished a big life goal after a long struggle to succeed.<br><br>In the moment when he (she) accomplished it, he (she) was overwhelmed with strong emotions. This is how he (she) reacted in the moment when he (she) accomplished his (her) goal.	이번 상황은 한 남성(여성)이 성공하기 위해 오랜 시간 노력하였지만 목표달성에 실패한 순간을 상상해주시기 바랍니다.<br><br>남성(여성)은 인생 목표 달성에 실패했을 때, 강렬한 감정에 빠졌습니다. 다음 사진은 목표달성에 실패한 순간 남성(여성)의 반응입니다.	In this scenario, we would like you to imagine a man (woman) who failed to accomplish a big life goal after a long struggle to succeed.<br><br>In the moment when he (she) failed, he (she) was overwhelmed with strong emotions. This is how he (she) reacted in the moment when he (she) failed to accomplish his (her) goal.	*M* = 4.57<br>*SE* = .22	*M* = 1.40<br>*SE* = .20	*M* = 2.10<br>*SE* = .21	*M* = 3.84<br>*SE* = .19
Reunified With Family	이번 상황은 한 남성(여성)이 오랜시간 떨어져 지냈던 가족과 다시 만난 순간을 상상해 주시기 바랍니다.<br><br>남성(여성)은 가족과 다시 만났을 때, 강렬한 감정에 빠졌습니다. 다음 사진은 가족과 만난 순간 남성(여성)의 반응입니다.	In this scenario, we would like you to imagine a man (woman) who was reunited with his (her) family after a long absence.<br><br>In the moment when he (she) saw his (her) family, he (she) was overwhelmed with strong emotions. This is how he (she) reacted in the first moment when he (she) finally was able to see his (her) family again.	이번 상황은 한 남성(여성)이 오랜시간 떨어져 지냈던 가족과 다시 만날 수 없다는 것을 알게 된 순간을 상상해 주시기 바랍니다.<br><br>남성(여성)이 가족과 만날 수 없다는 것을 알았을 때, 강렬한 감정에 빠졌습니다. 다음 사진은 가족과 만나지 못한다는 것을 알게 된 순간 남성(여성)의 반응입니다.	In this scenario, we would like you to imagine a man (woman) who was not able to reunite with his (her) family after a long absence.<br><br>When he (she) found out he (she) was not able to see her family, he (she) was overwhelmed with strong emotions. This is how he (she) reacted in the first moment when he (she) found out that he (she) would not be able to reunite with his (her) family.	*M* = 4.29<br>*SE* = .22	*M* = 1.68<br>*SE* = .22	*M* = 1.82<br>*SE* = .24	*M* = 3.78<br>*SE* = .24
Seeing Favorite Celebrity	이번 상황은 한 남성(여성)이 제일 좋아하는 연예인을 만난 순간을 상상해 주시기 바랍니다.<br><br>남성(여성)이 제일 좋아하는 연예인을 만났을 때, 강렬한 감정에 빠졌습니다. 다음 사진은 제일 좋아하는 연예인을 만난 순간 남성(여성)의 반응입니다.	In this scenario, we would like you to imagine a fan who was able to see his (her) favorite celebrity.<br><br>In the moment when he (she) saw his (her) favorite celebrity, he (she) was overwhelmed with strong emotions. This is how he (she) reacted in the first moment when he (she) saw his (her) favorite celebrity.	이번 상황은 한 남성(여성)이 제일 좋아하는 연예인을 만날 수 없게 된 순간을 상상해 주시기 바랍니다.<br><br>남성(여성)이 가장 좋아하는 연예인을 만날 기회를 놓쳤을 때, 강렬한 감정에 빠졌습니다. 다음 사진은 제일 좋아하는 연예인을 만날 기회를 놓친 순간 남성(여성)의 반응입니다.	In this scenario, we would like you to imagine a fan who was not able to see his (her) favorite celebrity.<br><br>In the moment when he (she) missed seeing his (her) favorite celebrity, he (she) was overwhelmed with strong emotions. This is how he (she) reacted in the first moment when he (she) missed seeing his (her) favorite celebrity.	*M* = 4.34<br>*SE* = .16	*M* = 1.63<br>*SE* = .19	*M* = 1.59<br>*SE* = .17	*M* = 3.45<br>*SE* = .20

* An independent sample rated to what extent each vignette would be considered a positive (or negative) event with two items (r =-.50).
Response options were: 1=*not at all positive (negative)* , 2=*a little bit positive (negative)* , 3=*moderately positive (negative)* , 4=*positive (negative)* , and 5=*extremely positive (negative)*
All vignetttes had significantly different positive and negative ratings, all p's < .001. Vignette type did not interact with item (p =.555), positive or negative context (p =.402), or item x context x vignette (p =.458).
